# The Future of Tissue-Targeted Lipid Nanoparticle-Mediated Nucleic Acid Delivery

**DOI:** 10.3390/ph15070897

**Published:** 2022-07-20

**Authors:** Ruvanthi N. Kularatne, Rachael M. Crist, Stephan T. Stern

**Affiliations:** Nanotechnology Characterization Laboratory, Cancer Research Technology Program, Leidos Biomedical Research, Inc., Frederick National Laboratory for Cancer Research Sponsored by the National Cancer Institute, Frederick, MD 21702, USA; ruvanthinilanga.kularatne@nih.gov (R.N.K.); cristr@mail.nih.gov (R.M.C.)

**Keywords:** lipid nanoparticles, drug delivery, therapeutic nucleic acids

## Abstract

The earliest example of in vivo expression of exogenous mRNA is by direct intramuscular injection in mice without the aid of a delivery vehicle. The current state of the art for therapeutic nucleic acid delivery is lipid nanoparticles (LNP), which are composed of cholesterol, a helper lipid, a PEGylated lipid and an ionizable amine-containing lipid. The liver is the primary organ of LNP accumulation following intravenous administration and is also observed to varying degrees following intramuscular and subcutaneous routes. Delivery of nucleic acid to hepatocytes by LNP has therapeutic potential, but there are many disease indications that would benefit from non-hepatic LNP tissue and cell population targeting, such as cancer, and neurological, cardiovascular and infectious diseases. This review will concentrate on the current efforts to develop the next generation of tissue-targeted LNP constructs for therapeutic nucleic acids.

## 1. Introduction

The earliest example of in vivo expression of exogenous mRNA was by direct intramuscular injection in mice without the aid of a delivery vehicle [1]. This appears to defy what is known about nucleic acids, being that they are large, polar and metabolically unstable drugs that do not cross cell membranes. However, since cytoplasmic delivery is essential for mRNA transcription and efficacy, clearly “naked” mRNA at a sufficient dose can be taken up by cells and expressed in vivo, at least to some degree. Later investigations identified saturable, nucleotide-specific uptake mechanisms that involve intracellular vesicles [2]. Regardless of existing mechanisms for direct nucleotide cellular uptake, delivery platforms offer stability, suppression of immunogenicity and dramatically improved cellular transfection.

## 2. LNP Chemistry, Formulation and Background

**Figure 1:**
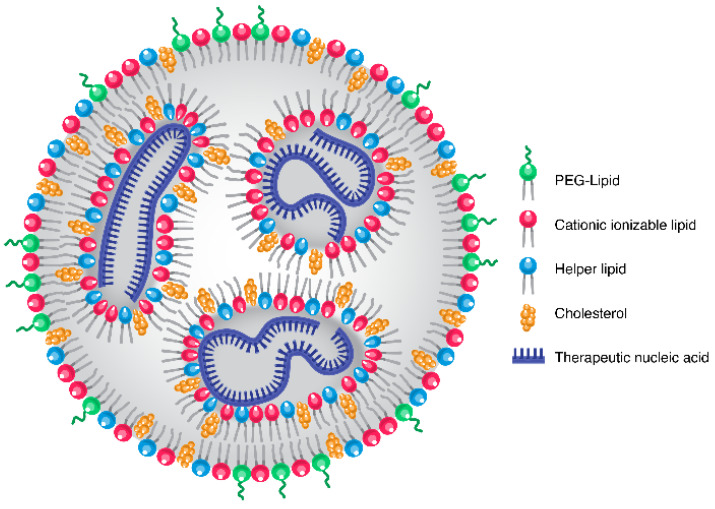
LNP Structure. The LNP interior contains electrostatically neutral inverted micelles, in which the negatively charged nucleic acid is surrounded by the ionizable lipid and other lipid components. The surface of the LNP is composed of a hydrophilic shell containing the PEG-lipid. (Figure adapted from Evers et al. [25]).

Formulating therapeutic nucleic acids into nanoparticles is of utmost importance to prevent degradation by nucleases upon administration and to enhance cellular uptake of these negatively charged entities. The current state of the art for therapeutic nucleic acid delivery is lipid nanoparticles (LNP), which are composed of cholesterol, a helper lipid, a PEGylated lipid and an ionizable amine-containing lipid (Figure 1) [3]. The cholesterol and helper lipids are important for the integrity of the LNP, while the PEGylated lipid provides colloidal stability as well as stealth properties to limit accumulation in the reticuloendothelial system (RES). The most important ingredient in this recipe is the ionizable amine-containing lipid, which is responsible for the complexation of nucleic acid. Importantly, this ionizable lipid is only protonated at non-physiological pH, pKa 6–7, which means the lipid is not charged in the circulation [4], which is important as cationic nanoparticles are notoriously toxic [5]. Upon cell uptake and lysosomal localization, the ionizable lipid is again charged at the low lysosomal pH, which, together with the unique conical features of the component lipids, assists in lysosomal escape and mRNA expression or siRNA gene silencing [4].

Alcohol dilution is the most commonly used method for LNP formulation, in which the nucleic acid payload is dispersed in an aqueous buffer (e.g., citrate, acetate, HEPES, malic acid buffers) at an acidic pH (pH ~ 3–5) and the excipients are dissolved in alcohol, ethanol being predominantly used, but *t*-BuOH is also used occasionally [6]. The ionic strength of the buffer varies from 10 mM to 100 mM, where lower ionic strength buffers are used for smaller RNA and pDNA and a higher concentration is used for larger RNA. During formulation, the aqueous and organic phases are generally combined at a volume ratio of 3:1 by either rapid mixing with a pipette or using microfluidic mixing. Downstream processing consists of either dialysis, tangential flow filtration or centrifuge filtration against PBS to remove ethanol and for buffer exchange.

Tissue targeting LNP can be obtained by introducing targeting ligands directly to the formulation in ethanol, chemically conjugating to the LNP surface, or by modifying the composition of the lipids in the formulation. Herein, active targeting refers to LNPs that contains a target-specific ligand in the formulation, whereas passive targeting refers to constructs lacking chemically conjugated targeting moieties.

Active targeting of LNP using antibodies was adopted by several groups, where a functionalized DSPE-PEG was introduced during LNP formulation at 12.5–25 mol% of total PEG, followed by chemically grafting the antibody [7,8,9]. For example, a simple amidation was used to conjugate αCD34 antibody to DSPE-PEG-carboxyl [8], and thiol-maleimide conjugation was employed to attach anti-CD4 antibody [9,10] and mAb specific for PECAM-1 [11] to DSPE-PEG-maleimide containing LNP. Caveolae targeted delivery to the lungs was achieved by conjugating Fab-C4 to DSPE-PEG-maleimide via a Diels-Alder reaction, where the Fab-C4 contains a cyclopentadiene lysine derivative to allow the Diels-Alder transformation [7]. A different strategy was utilized by Goswani et al. where the targeting ligand was attached to cholesterol instead of PEG-lipid. Here, α-mannose containing an aminopropyl succinate spacer was conjugated to cholesterol via an amide bond and formulated into LNP to deliver saRNA to dendritic cells [12]. An example of introducing a targeting ligand directly into the formulation is whereby DSPE-PEG2000-mannose was incorporated into the formulation at 2.5 mol% (at 3 mol% total PEG-lipid) to allow selective delivery of LNP to liver sinusoidal endothelial cells [13].

Passive targeting is governed primarily by the size and charge of the LNP, which is acquired through changes in the molar compositions of the four types of lipids used in the formulation. One such example is the use of increasing amounts of DMG-PEG2000 from 0.004 µmol to 0.12 µmol to reduce the LNP size from 200 nm to 30 nm. The latter was shown to have enhanced cellular uptake by CD+ dendritic cells in lymph nodes [14]. The same report showed the use of CHEMS at ~20 mol% to obtain negatively charged LNP to further enhance cellular uptake. Replacing traditional linear PEG-lipids with 3% Tween 20, which contains three PEG chains and a single lipid chain, Zukancic et al. was able to demonstrate targeted delivery of pDNA LNP to draining lymph nodes, however at the expense of reduced encapsulation efficiency, ~50% [15]. On occasion, an additional lipid is introduced to achieve passive targeting, which has been termed selective organ targeting (SORT) lipids [16,17]. These SORT lipids are introduced to the LNP formulation by dissolution in THF or ethanol at different molar ratios before mixing with the RNA to obtain liver, spleen and lung targeting [17,18,19,20]. Lipid composition and the type of ionizable lipid used in the formulation have a greater impact on pDNA LNP transfection, with different ratios of DODAP and DOPE in the formulation [21] and changing the ionizable lipid from DLin-MC3-DMA to DLin-KC2-DMA [22] increasing transfection in the spleen, while uptake of these LNP was greatest in the liver [21,22].

Although many LNPs are currently in preclinical and clinical development, only three have been approved/authorized by the US Food and Drug Administration (FDA) for clinical use. These are Comirnaty^®^ SARS-CoV-2 mRNA vaccine by BioNTech/Pfizer, mRNA-1273 SARS-CoV-2 mRNA vaccine by Moderna and Onpattro^®^ transthyretin siRNA for hereditary amyloidosis by Alnylam [23]. By way of their rapid regulatory review and enormous clinical impact, the market approval of the LNP-based mRNA vaccines, in particular, will facilitate the translation of future nanomedicine products [23]. This truth has already been recognized by the savvy financial markets, with record investment flowing into nanomedicine startups [24], especially vaccine companies.

## 3. Inherent LNP Liver Tropism

The liver is the primary organ of LNP accumulation following intravenous administration and is also observed to varying degrees following intramuscular and subcutaneous routes, with larger LNP having less liver uptake [26]. For example, after intramuscular administration of an mRNA LNP vaccine to mice, branched DNA analysis of tissue mRNA identified muscle > lymph node > liver > spleen > testis as the primary organs of LNP accumulation in descending order [27]. Interestingly, mRNA LNP uptake does not necessarily correlate with mRNA protein expression [26]. The cell populations involved in liver uptake are dependent upon the underlying uptake mechanism. A common mechanism of uptake for most nanoparticles, including LNP, is scavenger receptor-mediated uptake into the hepatic Kupffer cells and sinusoidal endothelium following opsonization by non-specific absorption of plasma proteins [28]. Alternatively, Akinc et al. discovered that adsorption of endogenous apolipoprotein E (ApoE) can direct the uptake of LNP to hepatocytes through interaction with the low-density lipoprotein receptor (LDLR) located in these cells [29]. These researchers characterized this uptake mechanism by using LDLR knockout mice and hepatocytes in vitro and went on to show that the *N*-acetylgalactosamine (GalNAc) ligand covalently attached to LNP could also target hepatocytes through the asialoglycoprotein receptor (Table 1). The utility of GalNAc-mediated hepatocyte targeting of LNP is demonstrated by the recent approval of Onpattro transthyretin siRNA for hereditary amyloidosis mentioned above. Clearly, delivery of nucleic acid to hepatocytes by LNP has therapeutic potential, but there are many disease indications that would benefit from non-hepatic LNP tissue and cell population targeting, such as cancer and neurological and cardiovascular disease.

Although reducing hepatic uptake of parenterally administered LNP has been a challenge, alterations in LNP composition and physicochemical characteristics have been shown to influence liver distribution (Table 1). The LNP surface charge has been shown to influence the liver accumulation of intramuscularly administered LNP, with negatively charged LNP having greater liver uptake [4]. Similarly, the inclusion of neutral lipids to LNP resulted in liver tropism following intravenous injection, while the addition of cationic lipids to net neutral LNP resulted in a shift to lung uptake and the addition of negatively charged lipids to net neutral LNP resulted in splenic uptake [17]. The addition of oxidized cholesterol to LNP shifted liver uptake away from hepatocytes and into the hepatic microenvironment, including Kupffer cells and hepatic endothelial cells [30]. The inclusion of constrained lipids in LNP, such as adamantyl phospholipids, has also been shown to target hepatic Kupffer cells [31]. Apart from charge and composition, size can also influence hepatocyte transfection, presumably due to the hepatic architecture, with narrow sinusoidal fenestration pores of ~100 nm [32]; LNP > ~200 nm dramatically diminishes hepatocyte transfection [33].

Alternatives to LNP modification have also been attempted to prevent liver accumulation. In one example, a liposome termed a “nanoprimer” was used to saturate Kupffer cell-mediated clearance 10 min prior to administration of LNP containing Cy5.5 labeled human erythropoietin mRNA or factor VII siRNA, resulting in increased systemic fluorescence at 1 h post-dose and increased erythropoietin or decreased factor VII protein expression at 48 h post-dose, respectively [34]. Clever mRNA modifications have also been made in an attempt to reduce off-target liver expression by the design of hepatic-selective, suppressive micro-RNA (miRNA) binding sites into the untranslated regions of the mRNA [35]. This would decrease hepatic translation regardless of hepatic LNP uptake. The incorporation of suppressive miRNA elements can also be used to limit mRNA expression to a certain cell type. Magadum et al. utilized a miRNA responsive expression scheme they named ‘specific modRNA translation system’ or SMRT, to limit mRNA LNP expression to cardiomyocytes. In the SMRT scheme, a suppressive cardiomyocyte-specific miRNA binding site for miR1-208 was incorporated into the untranslated region of an mRNA coding for a negative-regulating protein, L7AE. Upon expression, the negative-regulating L7AE protein prevents the translation of the second mRNA of interest. However, in the case of cardiomyocyte-specific miRNA binding to the L7AE mRNA, the L7AE expression is suppressed and the second mRNA is translated [36].

**Table 1:** Tissue-targeted LNP. (DLS, dynamic light scattering; i.m., intramuscular; i.v., intravenous; s.c., subcutaneous; i.d., intradermal; i.p., intraperitoneal; r.o., retroorbital; PDI, polydispersity index; ZP, zeta potential).

LNP Targeting Components or Properties	Physicochemical Properties (DLS Size, PDI and Zeta Potential)	Route of Administration	Payload and Indication	Model	Tissue/Cell Type Specificity	Ref.
50 mol% 1,2-dioleoyl-3-trimethylammonium-propane (DOTAP)	113 nm 0.22 PDI −0.52 mV	i.v.	human Erythropoietin, mouse Interleukin-10, mouse Klotho, Luciferase and Cre mRNA, and Cas9 mRNA/sgTom1 *	18–20 g male C57BL/6 mice; (age and sex not specified) B6.Cg-Gt(ROSA)^26Sortm9(CAG-tdTomato)Hze^/J mice (also known as Ai9 or Ai9(RCL-tdT) mice	hepatocyte uptake	[17]
30 molar% 1,2-dioleoyl-*sn*-glycero-3-phosphate (18PA)	142 nm 0.13 PDI −2.11 mV	i.v.	human Erythropoietin, mouse Interleukin-10, mouse Klotho, Luciferase and Cre mRNA, and Cas9 mRNA/sgPTEN and Cas9 mRNA/sgTom1 *	18–20 g male C57BL/6 mice; (age and sex not specified) B6.Cg-Gt(ROSA)^26Sortm9(CAG-tdTomato)Hze^/J mice (also known as Ai9 or Ai9(RCL-tdT) mice	hepatocyte uptake	[17]
20 molar% 1,2-dioleoyl-3-dimethylammonium-propane (DODAP)	12 nm 0.18 PDI (ZP not specified)	i.v.	human Erythropoietin, mouse Interleukin-10, mouse Klotho, Luciferase and Cre mRNA, and Cas9 mRNA/sgPCSK9 and Cas9 mRNA/sgTom1 *	18–20 g male C57BL/6 mice; (age and sex not specified) B6.Cg-Gt(ROSA)^26Sortm9(CAG-tdTomato)Hze^/J mice (also known as Ai9 or Ai9(RCL-tdT) mice	hepatocyte uptake	[17]
50:10:38.5:1.5% mole ratios DLin-KC2 DMA:DSPC:Cholesterol: DMG-PEG2000, with N:P molar ratio = 2, imparting negative charge	75 nm (PDI not specified) −10 mV	i.m.	Luciferase mRNA *	8-week-old female Balb/c mice	greater hepatic distribution following i.m. administration	[4]
50:23.5:6.5:20% mole ratios 7C1 **:C14PEG2K:18:1 Lyso PC 60:10:25:5% mole ratios 7C1 **:C14PEG2K:DOPE	20–200 nm (PDI not specified) (ZP not specified)	i.v.	ICAM-2 siRNA, Cre mRNA, CRISPR-Cas9 mRNA and ICAM-2 sgRNA *	5–12-week-old (sex not specified) LSL-Tomato, C57BL/6J, and constitutive SpCas9 mice	lung, spleen, liver and kidney endothelial cell uptake	[37]
Endogenous absorption of apoE to neutral LNP	64.5 nm (PDI not specified) (ZP not specified)	i.v.	Factor VII siRNA *	6–8-week-old female C57Bl/6, ApoE−/− and LDLR−/− mice	hepatocyte uptake	[29]
*N*-acetylgalactosamine (GalNAc) ligand	69.4 nm (PDI not specified) (ZP not specified)	i.v.	Factor VII siRNA *	6–8-week-old female C57Bl/6 and ASGR2−/− mice	hepatocyte uptake	[29]
Plasmalemma vesicle-associated protein (PV1)	70 nm, 0.104 PDI and 160 nm, 0.150–0.240 PDI (ZP not specified)	i.v.	Luciferase mRNA, Cy5-mRNA *	5-week-old female Balb-c mice	lung uptake	[7]
Anti-Ly6c mAbs	70 nm (PDI not specified) (ZP not specified)	i.v.	Luciferase or IL-10 mRNA; treatment of inflammatory bowel disease	Colitis was induced in: 12-week-old female C57BL/6 mice using dextran sodium sulfate	leukocyte uptake	[38]
15–20 mol% C18PEG2000:80 mol% 7C1 **:0.1–10 mol% cholesterol	45–50 nm <0.2 PDI (ZP not specified)	i.v.	ICAM-2 siRNA, ICAM-2 targeting sgRNA *	5–12-week-old (sex not specified) C57BL6/j and constitutive SpCas9 mice	bone marrow endothelial cell	[39]
Anti-CD4 antibody	129 nm 0.12 PDI −10 mV	i.v.	Cy5-labeled siRNA and CD45 siRNA *	6–8-week-old (sex not specified) C57BL6/j mice	T cells	[9]
Anti-CD4 antibody	88 nm 0.1 PDI (ZP not specified)	i.v.	Cre recombinase-encoding mRNA *	(age not specified) (sex not specified) Ai6 (RCL-ZsGreen) mice on C57BL/6J	Splenic and lymph node T cells	[10]
Adamantane-constrained lipid	20–200 nm 0.20–0.23 PID (ZP not specified)	i.v.	GFP siRNA *	5–8-week-old female C57BL/6-Tg(UBC-GFP)30Scha/J, ‘GFP mice’	splenic T cells	[40]
Anti-CD29 antibody	66–75 nm 0.10–0.16 PDI (ZP not specified)	i.v.	PLK1 siRNA; treatment of disseminated bone marrow mantle cell lymphoma xenograft	8-week-old female C.B-17/IcrHsd-Prkdc scid mice	mantle cell lymphoma	[8]
Cholesterol oleate	22–115 nm (PDI not specified) (ZP not specified)	i.v.	ICAM-2 siRNA, GFP-targeted sgRNA *	5–8-week-old female C57BL/6J and C57BL/6-Tg(UBC-GFP)30Scha/J, ‘GFP mice’	hepatic endothelial cells	[41]
~30 nm, negatively charged LNP	34 nm 0.242 PDI −12 mV	s.c.	DiD-labeled LNP (no nucleic acid) *	7–9-week-old female C57BL/6J mice	CD8+ dendritic cells/lymph node	[14]
35:5:55:5% mole ratios 7C1 **:Cholesterol:C14PEG2000:DOTAP	40 nm (PDI not specified) (ZP not specified)	nebulization	Therapeutic membrane-anchored FI6 antibody mRNA, H1N1 influenza model	6–8-week-old female BALBc mice	lung	[42]
~150 nm size, ~0.5% PEG density	150 nm <0.1 PD (ZP not specified)	intravitreal and subretinal injection	Cre, mCherry, luciferase mRNA *	1–6 months old male and female Albino BALB/c, Ai9, apoE−/−, Mertk−/− and C57BL6 mice	optic nerve, trabecular meshwork, retinal pigment epithelium, Muller glia	[43]
Ionizable lipids with low pKa and unsaturated hydrocarbon chains	83–229 nm 0.09–0.28 PDI (ZP not specified)	subretinal injection	Luciferase, EGFP, mCherry mRNA *	1–4 months old male and female Albino BALB/c mice	retinal pigment epithelium	[44]
Oxidized cholesterol	~80 nm 0.16 PDI (ZP not specified)	i.v.	Cre mRNA *	5–8-week-old (sex not specified) Ai14 Lox-Stop-Lox-tdTomato and C57BL/6J mice	hepatic endothelial and Kupffer cells	[30]
Anti PECAM-1 antibody	103 nm 0.195 PDI −4.1 mV	i.v.	Luciferase mRNA *	(age not specified) (sex not specified) C57BL/6 mice	lung vascular endothelial and immune cells	[11]
Adamantyl-constrained lipid	100 nm (PDI not specified) (ZP not specified)	i.v.	Cre mRNA *	(age not specified) (sex not specified) Ai14 Lox-Stop-Lox-tdTomato mice	hepatic Kupffer cells	[31]
ApoE opsonization	55 nm 0.058 PDI (ZP not specified)	intracranial	PTEN, luciferase and GRIN1 siRNA *	26–30-day-old (sex not specified) Sprague Dawley rats	CNS neurons	[45]
CH6 osteoblast-specific aptamer	84 nm (PDI not specified) (ZP not specified)	i.v.	osteogenic pleckstrin homology domain-containing family O member 1 (*Plekho1*) siRNA; treatment of impaired bone formation (e.g., osteoporosis)	6-month-old female Sprague Dawley rats	osteoblasts	[46]
Mannose-cholesterol	~140 nm >0.2 PDI (ZP not specified)	i.d.	Influenze hemagglutanin saRNA; H1N1 influenza vaccine	6−8-week-old female BALB/c mice	dendritic cells	[12]
Mannose-PEG-DSPE	~100 nm (PDI not specified) (ZP not specified)	i.v.	Cre mRNA and FVIII siRNA *	7–10-week-old female C57BL/6 mice; 8-week-old (sex not specified) Lox-Stop-Lox-tdTomato	hepatic endothelial cells	[13]
EGFR-antibody	79 nm 0.085 PDI 7.7 mV	i.p.	Cas9 mRNA, (polo-like kinase) PLK1 sgRNA; disseminated ovarian cancer	8-week-old female Hsd: Athymic Nude-Foxn1nu mice with OV8 ovarian cancer peritoneal xenograft	disseminated ovarian cancer	[47]
DEC205-antibody	90–130 nm 0.12–0.20 PDI (ZP not specified)	r.o.	CD40, CD80 and CD86 siRNA	6–12-week-old (sex not specified) C57BL/6 mice, inhibition of mixed lymphocyte response to LPS	CD8 alpha+ dendritic cells	[48]
CD4-antibody	88 nm 0.1 PDI (ZP not specified)	i.v.	Luciferase and Cre mRNA *	(age not specified) male and female C57BL/6 and Ai6 (RCL-ZsGreen) mice on C57BL/6J background	CD4+ T cells	[10]
CD5-antibody	80 nm 0.02–0.06 PDI (ZP not specified)	i.v.	CAR mRNA against fibroblast activation protein and Cre mRNA, cardiac fibrosis prevention	(age not specified) (sex not specified) C57BL/6NAi6 Cre-reporter mice (Rosa26^CAG-LSL-ZsGreen^)	CD5+ T cells	[49]

* Experimental system, no therapeutic indication evaluated. ** 7C1 is a novel ionizable lipid; refer to publication for structure.

## 4. Non-Hepatic LNP Targeting

Several groups have used in vivo screening systems to select LNP compositions that target-specific tissues/cells without the use of targeting ligands. In particular, Dahlman’s laboratory at the Georgia Institute of Technology has pioneered the use of an in vivo screen that correlates a unique LNP “DNA barcode” with siRNA delivery and function [39]. The technique identifies reduced protein expression resulting from siRNA knockdown in the tissue of interest by flow cytometry and then sequences the cells to identify the corresponding DNA barcode of the LNP responsible. Utilizing this method, over 100 LNP of varying composition were screened simultaneously for bone marrow endothelial cell (BMEC) transfection with an ICAM-2 siRNA payload, identifying LNP with 15–20 mol% C18PEG2000/80 mol% 7C1 helper lipid/0.1–10 mol% cholesterol as BMEC targeted. They then went on to show that an example of this BMEC-targeting LNP, ‘BM1′, could also deliver sgRNA in a constitutively expressed SpCas9 model, demonstrating the flexibility of this delivery platform.

LNP targeting of immune cell populations is of particular interest for the purposes of immunomodulation and vaccine delivery. While intramuscular injection of untargeted mRNA LNP vaccines results in significant accumulation in antigen-presenting cell (APC) populations at local lymph nodes, there is substantial accumulation in the muscle itself, as well as other tissues such as liver, spleen, bone marrow and testes [27]. The potential negative consequences of this off-target distribution are presently unknown. In order to increase vaccine efficacy as well as decrease potential risks of off-target distribution, researchers have tried to improve LNP distribution to lymph nodes and APC. As an example, Nakamura et al. evaluated the effect of size and charge on lipid nanoparticle lymph node tropism of LNP prepared by the popular microfluidic mixing technique [41]. They identified small (~30 nm), negatively charged LNP as having greatly superior lymph node dendritic cell distribution in comparison to larger (100–200 nm), neutral or cationic LNP [14]. This agrees with liposome studies by Kranz et al., who identified negatively charged liposomes as having greater lymphatic dendritic cell tropism [50]. Active targeting has also been utilized to target vaccines to APC. Incorporation of mannose conjugated cholesterol in an influenza hemagglutinin saRNA LNP to target the APC mannose receptor (CD206) resulted in enhanced dendritic primary cell uptake in vitro and a more rapid immune response in vivo following i.d. administration, with higher antibody titers and greater antigen-specific splenic CD4+ and CD8+ T cells [12]. The incorporation of a mannose targeting ligand has also been shown to target LNP to hepatic sinusoidal endothelial cells [13].

LNP has been targeted to T cells with surface conjugated anti-CD4 antibodies, resulting in uptake by T cells in the blood and lymphatic system following intravenous administration [9,10]. The LNP with conformationally constrained lipids was also found to target splenic T cells following intravenous administration [40]. Coating of antibodies on the surface of LNP incorporating an Fc binding lipid allowed for in vivo targeting of various immune cells, including macrophages, Treg, T helper, CTL, B cells and monocytes, corresponding to coating with anti-CD45, -CD25, -CD4, -CD8, -CD19 and -CD11b antibodies, respectively [8].

Lung delivery of nucleic acids has the potential for the treatment of a wide range of respiratory diseases, such as genetic defects such as cystic fibrosis and infectious diseases such as the common flu. In addition to systemic administration of lung-targeted LNP, direct inhalation of nebulized LNP has been attempted [7,17,42]. In the case of nebulization, researchers found high molar percentages of both PEG and cationic helper lipid improved lung transfection [42]. Administration of a nebulized 40 nm LNP with 55 and 5 molar% C14PEG2000 and DOTAP, respectively, delivered a payload of membrane-anchored FI6 antibody mRNA and protected against a lethal dose of influenza H1N1 in a murine model. Systemically administered LNP has also been targeted to the lung vasculature by conjugation of platelet endothelial cell adhesion molecule-1 (PECAM-1) antibodies to the LNP surface [11].

The eye is also of interest for LNP delivery for the treatment of such conditions as retinal degeneration. The eye benefits from being accessible for direct administration by topical, subretinal, intravitreal and suprachoroidal administration routes [51]. At this time, only viral vector-based oligonucleotide therapies are approved for the treatment of ocular diseases, such as Luxturna for inherited retinal dystrophy [51], but advancements are being made in LNP oligonucleotide delivery systems. Patel et al. identified LNP containing ionizable lipids with low pKa and unsaturated hydrocarbon chains as having the greatest retinal pigment epithelium (RPE) transfection following subretinal injection [44]. These researchers went on to discover that larger LNP, ~150 nm, having a lower PEG density of 0.5 mol% had greater transfection of the RPE following subretinal administration, and following intravitreal administration, had greater transfection of Muller glia, optic nerve and trabecular meshwork [43]. ApoE absorption and subsequent LDLR-mediated uptake, as well as phagocytosis, were not involved in the observed LNP RPE transfection.

LNP targeting of oligonucleotides to the CNS has potential for the treatment of neurological diseases as well as providing a tool for understanding brain function through manipulation of protein expression. Since ApoE is produced by astroglia and LDLR is found on neurons, LNP can be used to target neurons in the CNS, similar to how hepatocytes are targeted systemically in an ApoE-dependent fashion [52]. Back in 2013, Rungta et al. demonstrated that intracranial administration of siRNA-LNP constructs to the brain could silence neuronal *N*-methyl-D-aspartate (NMDA) receptors locally or regionally when administered by intracortical or intracerebroventricular injection, respectively [45]. They demonstrated that neuronal LNP uptake was ApoE-dependent by observing that LNP uptake into rat primary neurons only occurred upon supplementation of the culture media with ApoE. The primary issue for LNP-mediated CNS delivery, however, is overcoming the blood–brain barrier (BBB). The only example we found in our literature review for a BBB-targeted LNP utilized an RNA aptamer targeting the C-C chemokine receptor type 5 (CCR5), and this construct was only evaluated in an in vitro model of the BBB, not in vivo.

## 5. Oncology and Immuno-Oncology

An area of great promise for the future of LNP is cancer, in particular vaccine immunotherapy [53,54]. In fact, both Moderna and BioNTech, developers of the FDA-approved SARS-CoV-2 vaccines, are investing in immuno-oncology to further utilize their mRNA delivery technologies. BioNTech has several mRNA cancer vaccine candidates in clinical trials, including BNT111, in phase 2 clinical trials for melanoma (NCT04526899), BNT113, in phase 2 for HPV16-positive head and neck cancers (NCT04534205), and BNT112 in phase 1/2 for prostate cancer (NCT04382898). These mRNA vaccines are being explored in combination with anti-PD-1 immune checkpoint inhibitors such as cemiplimab and pembrolizumab [55]. Though, these formulations use BioNTech’s FixVac platform technology (RNA-lipoplex delivery vehicles), not the LNP architecture as shown in Figure 1. Lipoplexes were one of the early delivery vehicles for mRNA targeting, although with the advantages lipid nanoparticles offer, such as increased stability and protection of the nucleic acid cargo, lipid nanoparticles are becoming a popular choice for the exploration of novel treatment strategies [53]. Additionally, in a recent interview, BioNTech CEO Uğur Şahin stressed the company’s commitment to developing strategies to combat cancer with its mRNA cancer vaccine technology [56].

Moderna is using its LNP technology for the delivery of mRNA cancer vaccines as well as immuno-oncology therapeutics [57]. The following two cancer vaccine candidates are currently in clinical trials: mRNA-4157, a personalized cancer vaccine in phase 2 clinical trials for the treatment of melanoma (NCT03897881), and mRNA-5671, a KRAS vaccine in phase 1 clinical trials for the treatment of pancreatic, colorectal and non-small cell lung cancers (NCT03948763) [58]. Both vaccines are being tested in combination with pembrolizumab. In the immuno-oncology space, Moderna has the following two formulations in Phase 1 clinical trials: mRNA-2752, an LNP encapsulating mRNA encoding OX40L, IL-23 and IL-36γ (NCT03739931, NCT02872025) and MEDI1191, an LNP encapsulating mRNA for IL-12 (NCT03946800) [58]. These formulations are being tested in combination with pembrolizumab and durvalumab, respectively.

Recently, Pfizer entered into an agreement with Acuitas Therapeutics to license their LNP technology for the development of several therapeutic and vaccine concepts [59]. There are already dozens of strategies in clinical trials for both cancers as well as infectious disease treatment and vaccine development [54]. Further, there are countless strategies in various phases of preclinical development. Oberli et al. have described a lipid nanoparticle-based mRNA vaccine that showed promise when tested in a B16F10 melanoma model in mice [60]. The vaccine, encoding for TRP2 and gp100 tumor-associated antigens, induced strong CD8 T cell activation and showed decreased tumor volume with increased animal survival in the highly aggressive cancer. In another example, Lee et al. used LNP for the delivery of tri-palmitoyl-S-glyceryl cysteine linked to a pentapeptide (termed Pam3), which is a known adjuvant of TLR1 and TLR2 [61]. In addition to enhanced CD8 T cell response, the Pam3-LNP showed superior tumor prevention in a mouse lymphoma model. Novel LNP-mRNA vaccines and treatments hold tremendous potential for future development, and with improved targeting strategies as described herein, the number of both preclinical and clinical studies is sure to multiply in the coming years.

## 6. Conclusions and Future Directions

**Figure 2:**
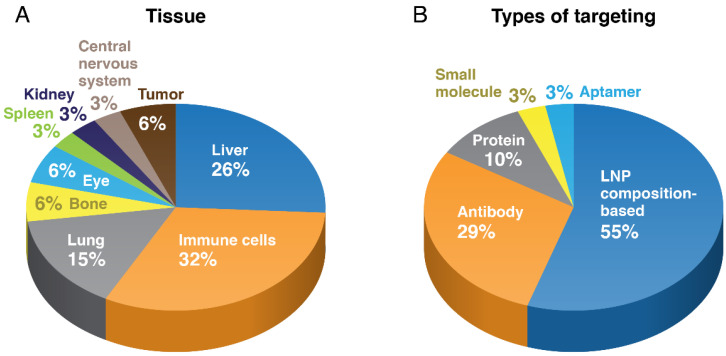
Trends in targeted LNP. The trends in LNP tissue target (**A**) and targeting mechanism (**B**) are displayed (from references in Table 1).

There are surprisingly few examples of tissue-targeted LNP, apart from targeting of the liver, lung and the immune system, as well as a few examples of actively targeted concepts (Figure 2). One of the primary obstacles to tissue targeting is the natural physiological barrier to tissue accumulation presented by the vascular endothelium, as most tissues apart from the liver and spleen are not fenestrated, having a continuous endothelium [62]. While tumor vasculature often contains openings in the endothelium, the passive accumulation of nanoparticles through these pores into the tumor tissue has been found to be highly variable and inefficient, largely due to inconsistent pore size and density and extracellular matrix and lymphatic blockage that creates back pressure that counters convective and diffusive movement into the pore [63]. Additionally, animal cancer models, primarily mice, are often not representative of the clinical case and this has resulted in a poor correlation between preclinical and clinical efficacy of nanomedicine formulations [64]. Studies reviewing nanomedicine tumor uptake in the preclinical literature have identified both poor absolute tumor uptake, averaging ~0.7% of the total dose, but also greater tumor vs. systemic drug exposure in comparison to conventional drug formulations [65,66]; these data would support the reduced toxicity of nanomedicines, which generally correlates with systemic exposure, but also reduced efficacy, which correlates to tumor exposure.

With this in mind, it is of interest for future targeted LNP research to concentrate on active targeting of the vascular endothelium itself, not only the tissue of interest, utilizing strategies such as receptor-mediated transcytosis and paracellular transport to breach the vascular barrier [67,68]. Selectivity of this endothelial targeting strategy must rely on tissue-selective receptor expression that may benefit from modern ligand-receptor identification techniques such as phage display. Phage display is a versatile tool that can be used to screen proteins, peptides or antibodies for interaction with cells, tissues or biomarkers, conducted in vitro, ex vivo, as well as in vivo [69,70]. In addition to the identification of more selective targeting ligands, there is also the requirement to simultaneously optimize transfection efficiency, which as mentioned above is also tissue-dependent [26].

An alternative to tissue targeting using LNP composition or the addition of a targeting ligand is the incorporation of cell membrane-derived components into LNP. Addition of cell membrane-derived components to make biological hybrid LNP constructs can utilize the cell membrane’s innate-stealth qualities to evade immune system recognition and homotypic features to target tissues and cells [71]. Common cell membrane-derived coatings used for stealth and tissue targeting are red blood cell (RBC) membranes, and cancer and platelet cell membranes, respectively [72,73,74,75]. This powerful targeting technique has been applied to patient-derived cancer cells (PDCC), with PDCC membrane-coated nanoparticles demonstrating PDCC-specific targeting preclinically in their respective xenograft models [73]. Similarly, cancer cell membrane-coated siRNA formulations have also been shown to selectively target in a homotypic fashion, in which only cancer cells of the membrane origin are targeted [76]. There is also the possibility of using a combination of active and biomimetic targeting approaches. For example, a cRGD-targeted, RBC membrane-coated polyplex was utilized for siRNA delivery to melanoma, with the RBC coating preventing opsonization and increasing circulation time [77]. Targeting ligands can also be engineered directly into the coating membrane. Park et al., for example, coated an mRNA polyplex with a mouse melanoma membrane engineered to express a viral fusion protein that enhances endosomal escape, dramatically improving transfection efficiency both in vitro and in vivo [78]. A more general targeting approach than cancer cell membrane homotypic targeting and active targeting are utilizing platelet membrane coatings, which have been shown to target a variety of disease states, including vascular disease, infections and cancer [75]. Utilizing this disease agnostic targeting approach, Zhuang et al. demonstrated the ability of a platelet membrane-coated survivan siRNA metallic nanoparticle to accumulate in and suppress the growth of an SK-BR-3 breast cancer xenograft [79].

Another potentially impactful application of LNP technology is modification to exploit stimuli-responsive properties. Internal (e.g., pH, enzyme and redox) and external (e.g., temperature, light, ultrasound and magnetic fields) stimuli have been explored for a variety of nanoparticle platforms [80,81,82,83,84], with Celsion Corporation’s ThermoDox arguably the most well-known example [85,86,87]. There are several reports in the literature using internal and external stimuli to afford the release of nucleic acid cargos. Miller et al. synthesized cationic lipoplexes incorporating matrix metalloproteinase-2 (MMP-2) and human leukocyte elastase (HLE) sensitive linkers, both of which are known to be upregulated in the tumor matrix, to demonstrate the targeted release of siRNA in vitro in several cell lines using luciferase knockdown experiments [88]. PEGylated lipids were modified to include the MMP-2 and HLE linkers, which when cleaved, effectively de-shield the nanoparticles of the PEG layer, promoting cellular uptake. In another example, Rabbitts et al. used external acoustic shock waves to enhance cellular uptake of mRNA lipoplex particles [89]. The lipoplex particle was engineered to take advantage of unique phase transitions that are triggered by the shock waves to promote cellular entry. Using fluorescently labeled GFP-modified mRNA, the lipoplexes were transfected into several cell lines and the fluorescence from the translated mRNA was quantified, with cells receiving the shock wave treatment showing a greater transfection efficiency. With appropriate modifications to either the lipid components or the nucleic acid cargo, LNP can also potentially take advantage of these internal and external release triggers to enhance tumor targeting.

## Data Availability

Data sharing not applicable.
